# The cone repair after Fontan procedure: Conversion from completed single-ventricle pathway after the Starnes procedure to biventricular physiology

**DOI:** 10.1016/j.xjtc.2025.05.030

**Published:** 2025-07-14

**Authors:** Jose P. Da Silva, Laura Seese, Laura Olivieri, Tarek Al-Sayed, Jacqueline Kreutzer, Mario Castro Medina, Victor O. Morell, Luciana Da Fonseca Da Silva

**Affiliations:** a Division of Pediatric Cardiothoracic Surgery, Department of Surgery, University of Pittsburgh Medical Center, UPMC Children's Hospital of Pittsburgh, Pittsburgh, Pa; b Division of Pediatric Cardiology, Department of Pediatrics, University of Pittsburgh Medical Center, UPMC Children's Hospital of Pittsburgh, Pittsburgh, Pa

**Figure:**
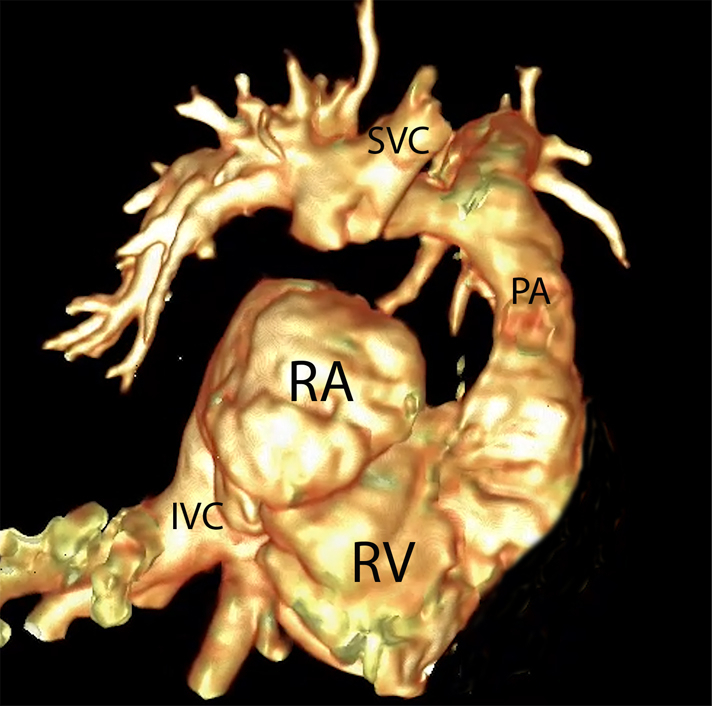
Conversion to 1.5-ventricle physiology after Starnes and Fontan procedures.

**Central Message**

The cone repair can rehabilitate the RV even after Fontan procedure in patients who previously underwent the Starnes procedure, establishing a new frontier on Ebstein anomaly treatment.

Critically ill neonates with Ebstein anomaly (EA) who present with a circular shunt or pulmonary atresia (PA) and severe right ventricular (RV) dilation benefit from RV exclusion through the Starnes procedure.^1^ Although lifesaving, this intervention leads to progressive RV involution and relegates patients to the single-ventricle pathway.^2^ Since 2019, we have changed this treatment paradigm by taking down the Starnes and using the cone operation with fenestrated atrial septal defect closure and RV outflow tract reconstruction to achieve biventricular repair.^3^ The cone repair after Starnes has only been applied to patients at the earlier stages of the single-ventricle pathway, either with an existing aortopulmonary shunt or bidirectional Glenn, due to concerns about age limitations for successful RV recruitment.^3^, ^4^, ^5^ Our evolving experience has demonstrated that the excluded RVs were consistently rehabilitated to provide adequate pulmonary circulation.^5^ We have applied the cone repair after Starnes approach to a symptomatic EA patient with Fontan physiology. Herein, we describe this novel strategy for biventricular conversion.

## Case Description

**Figure 1:**
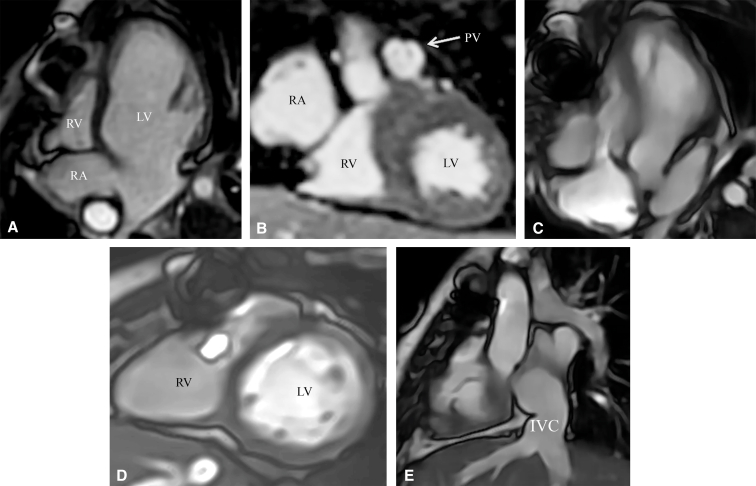
Preoperative and 6-month postoperative cardiac magnetic resonance imaging. A and B, Preoperative studies in 4-chamber and cross-section views, respectively, showing a small right ventricle (RV) beneath the Starnes patch with an RV end-diastolic volume (RVEDV) of 25 mL/m^2^, right ventricle ejection fraction (RVEF) of 10%, and a trileaflet pulmonary valve. C and D, Postoperative studies in 4-chamber and cross-section views, respectively, showing RVEDV of 56 mL/m^2^ and RVEF of 36%. E, Shows the IVC-RA connection. *LV*, Left ventricle; *PV*, pulmonary valve; *IVC*, inferior vena cava.

Our patient is a 7.9-year-old girl, an outside hospital referral, with severe EA and PA; she underwent a Starnes procedure as a neonate, then a Glenn operation, and a Fontan completion. Her mother noticed that over the past year she was fatigued and unable to keep up with her peers. A cardiac magnetic resonance imaging scan showed a small RV with severe dysfunction beneath the Starnes patch (Figure 1). Her heart catheterization confirmed a well-sized trileaflet pulmonary valve with severe supravalvular narrowing. The left ventricle was normal. She had Fontan-associated liver disease and concern for Alagille syndrome. A liver biopsy was reassuring. The mother gave informed written consent to publish; institutional review board approval was not required.

### Operative Technique

**Figure 2:**
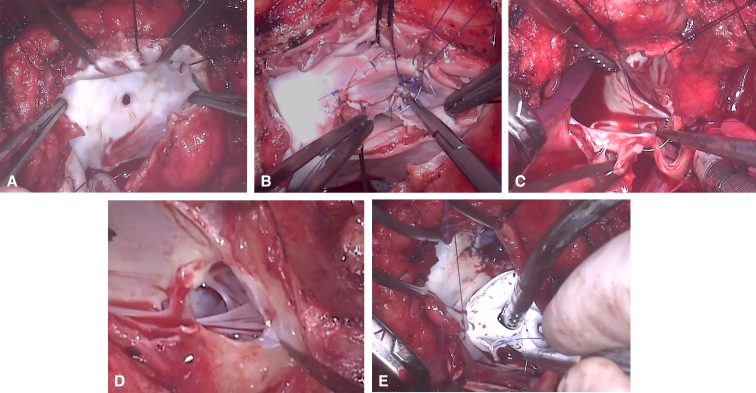
Operative steps of the Starnes takedown and cone repair after Fontan completion. A, The right atrium is widely opened, exposing the fenestrated Starnes patch, which was severely adherent to the underlying tricuspid valve tissue requiring careful dissection for removal. B, After mobilization of the severely displaced leaflets, the tricuspid valve was constructed in a *cone shape* and attached to the normal tricuspid annulus level. C, The inferior vena cava is reattached to the inferior aspect of the right atrium. D, The pulmonary valve (PV) is repaired by performing an incision at the pulmonary artery anterior wall to expose the pulmonary supra-valvar stenosis and the PV itself, we excised the stenotic area, made an incision toward the right PV sinus, and another incision toward the left sinus. The left Valsalva sinus was enlarged using fresh autologous pericardium patch. The main pulmonary artery and the right Valsalva sinus were enlarged using a polytetrafluoroethylene (PTFE) patch. E, The atrial septal defect is closed using a 0.4-mm fenestrated PTFE patch (4-mm fenestration).

After aortic and bicaval cannulation, systemic hypothermia, cardioplegia arrest, and ventricular venting through the atrial septal defect, we proceeded with the operation (Figure 2, Video 1). The tricuspid valve was extremely rotated with severe septal and inferior leaflets tethering. We made an incision at the anterior leaflet proximal attachment at 11 o'clock and extended toward the inferior leaflet. Then, the incision was extended counterclockwise until reaching the inferior aspect of the ventricular septum. We sectioned the anterior papillary muscle attached to the RV outflow tract area, rotated medially, and sutured to the septal leaflet superior distal edge. The extracardiac conduit was extracted, except for a short stump near the right PA, which was oversewn to preserve the Glenn anastomosis. We connected the native inferior vena cava tissue to the right atrium. After decannulation, the patient had multiple episodes of sinus bradycardia and supraventricular arrhythmias requiring central venoarterial extracorporeal membrane oxygenation support.

## Results

After the cone procedure, the intraoperative echocardiogram showed no TV stenosis with a mean diastolic gradient of 2.1-mm Hg, mild TV regurgitation, and moderate RV dysfunction. There was no PV stenosis or regurgitation. The direct measurement of the right atrial mean pressure was 9 mm Hg. She was decannulated from extracorporeal membrane oxygenation after 48 hours when she was in normal sinus rhythm. She was extubated the following day and discharged from the hospital on day 11. At her 6-month postoperative evaluation, she presented with no symptoms and oxygen saturation of 98%. Her tricuspid valve presented mild stenosis and no regurgitation, her RV volume had doubled, and its function significantly improved compared with early postoperative data (Figure 1).

## Discussion

Although the cone repair after Starnes has been applied to earlier stages of the single-ventricle pathway, this is the first cone after Starnes that was performed after a Fontan completion. We offered this procedure to improve symptoms, rehabilitate the RV, and partially takedown the Fontan circuit. Preserving the Glenn anastomosis for reasonable pulmonary perfusion is important until RV rehabilitation can support the full systemic venous return. We expect initial oxygen saturations in the high 80s, which gradually normalize as the RV remodels. Our patient's remodeling allowed for doubling the size of her RV in 6 months and demonstrates the potential for RV recovery even after Fontan completion.

We reported that patients who have undergone a cone repair after a Starnes can achieve competency of the tricuspid valve and good RV function at midterm follow-up.^5^ Current recommendations are that all patients with prior Starnes be evaluated for biventricular conversion with a cone repair.

Many patients who progress to the Fontan stage of the single-ventricle pathway will ultimately develop significant morbidities that limit transplant-free survival. The postoperative low right atrial pressure observed in our patient suggests that applying the cone procedure to convert the univentricular to 1.5-ventricle physiology may prevent the complications of the Fontan procedure. It removes the passive flow to the lungs by creating pulsatile flow through the subpulmonic ventricle that may relieve hepatic and intestinal venous congestion. This report demonstrates the potential for the functional RV to be recruited and perform well following a cone procedure in Fontan patients with a long-standing excluded RV. From our data on the cone repair after the Starnes procedure,^5^ the conversion from Fontan to 1.5-ventricle is viable in EA patients with growth potential who possess workable tricuspid valve leaflet tissues and an RV diastolic volume ≥15 mL/m^2^ on magnetic resonance imaging, particularly those facing an unfavorable clinical course after Fontan completion. Children and adults with little tricuspid valve leaflet tissue and fibrous RV myocardium are very unlikely to benefit from this procedure. However, adults having a reasonable RV size due to conditions allowing forward flow may be considered for this procedure.

## Conclusions

The cone repair after Fontan with a prior Starnes procedure is feasible, expanding the frontiers of EA treatment; however, postoperative RV remodeling patterns in this group of patients need to be further investigated before its widespread surgical application.

## Conflict of Interest Statement

The authors reported no conflicts of interest.

The *Journal* policy requires editors and reviewers to disclose conflicts of interest and to decline handling or reviewing manuscripts for which they may have a conflict of interest. The editors and reviewers of this article have no conflicts of interest.

## Supplementary Data

Video 1 Intraoperative video demonstrating the Starnes patch takedown, the cone repair of tricuspid valve, fenestrated atrial septal defect closure, the right ventricular outflow tract reconstruction, and Fontan takedown. The Glenn anastomosis was preserved. Video available at: https://www.jtcvs.org/article/S2666-2507(25)00273-1/fulltext.
