# Supplementary figures and images for: The cone repair after Fontan procedure: Conversion from completed single-ventricle pathway after the Starnes procedure to biventricular physiology
Source: JTCVS Tech. 2025 Jul 14;33:195–8. doi: 10.1016/j.xjtc.2025.05.030 (PMC12529691; doi:10.1016/j.xjtc.2025.05.030)

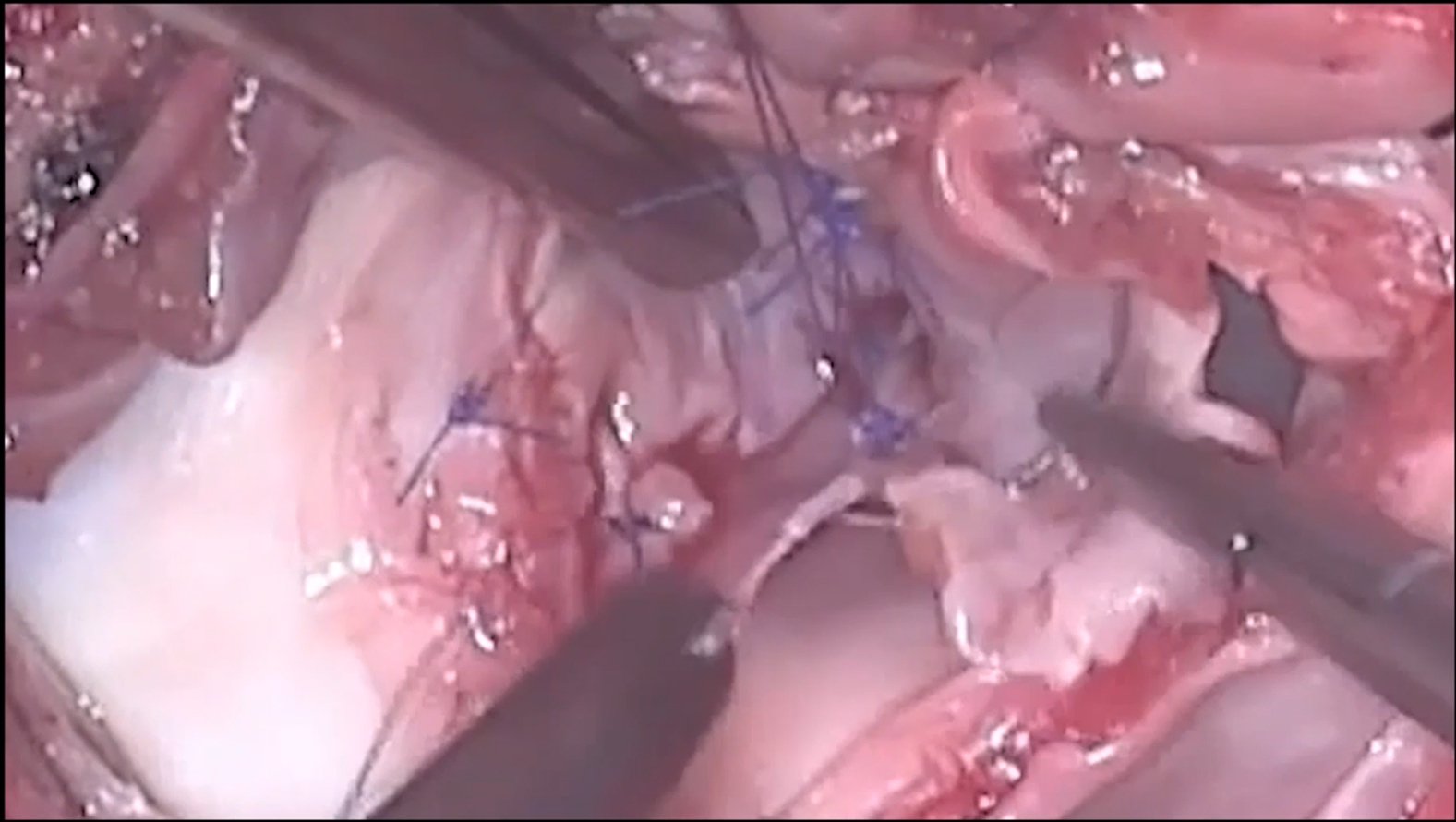

Supplement: Video 1 — Intraoperative video demonstrating the Starnes patch takedown, the cone repair of tricuspid valve, fenestrated atrial septal defect closure, the right ventricular outflow tract reconstruction, and Fontan takedown. The Glenn anastomosis was preserved. Video available at: https://www.jtcvs.org/article/S2666-2507(25)00273-1/fulltext. [file fx2.jpg]
